# Deep Learning–Based Multimodal Data Fusion: Case Study in Food Intake Episodes Detection Using Wearable Sensors

**DOI:** 10.2196/21926

**Published:** 2021-01-28

**Authors:** Nooshin Bahador, Denzil Ferreira, Satu Tamminen, Jukka Kortelainen

**Affiliations:** 1 Faculty of Information Technology and Electrical Engineering University of Oulu Oulu Finland

**Keywords:** deep learning, image processing, data fusion, covariance distribution, food intake episode, wearable sensors

## Abstract

**Background:**

Multimodal wearable technologies have brought forward wide possibilities in human activity recognition, and more specifically personalized monitoring of eating habits. The emerging challenge now is the selection of most discriminative information from high-dimensional data collected from multiple sources. The available fusion algorithms with their complex structure are poorly adopted to the computationally constrained environment which requires integrating information directly at the source. As a result, more simple low-level fusion methods are needed.

**Objective:**

In the absence of a data combining process, the cost of directly applying high-dimensional raw data to a deep classifier would be computationally expensive with regard to the response time, energy consumption, and memory requirement. Taking this into account, we aimed to develop a data fusion technique in a computationally efficient way to achieve a more comprehensive insight of human activity dynamics in a lower dimension. The major objective was considering statistical dependency of multisensory data and exploring intermodality correlation patterns for different activities.

**Methods:**

In this technique, the information in time (regardless of the number of sources) is transformed into a 2D space that facilitates classification of eating episodes from others. This is based on a hypothesis that data captured by various sensors are statistically associated with each other and the covariance matrix of all these signals has a unique distribution correlated with each activity which can be encoded on a contour representation. These representations are then used as input of a deep model to learn specific patterns associated with specific activity.

**Results:**

In order to show the generalizability of the proposed fusion algorithm, 2 different scenarios were taken into account. These scenarios were different in terms of temporal segment size, type of activity, wearable device, subjects, and deep learning architecture. The first scenario used a data set in which a single participant performed a limited number of activities while wearing the Empatica E4 wristband. In the second scenario, a data set related to the activities of daily living was used where 10 different participants wore inertial measurement units while performing a more complex set of activities. The precision metric obtained from leave-one-subject-out cross-validation for the second scenario reached 0.803. The impact of missing data on performance degradation was also evaluated.

**Conclusions:**

To conclude, the proposed fusion technique provides the possibility of embedding joint variability information over different modalities in just a single 2D representation which results in obtaining a more global view of different aspects of daily human activities at hand, and yet preserving the desired performance level in activity recognition.

## Introduction

It is a proven fact that chronic diseases including obesity, diabetes, and metabolic disorders are highly correlated with eating behavior and regarding the importance of this issue, application of wearable sensors for capturing eating-related activities has been widely studied in the literature [1-6]. These studies can be categorized into 3 groups, including food intake detection, food type classification, and food content estimation. Among these groups, food intake detection has been considered as the first phase in food intake monitoring, and studies around it mainly focused on detecting chewing activity (acoustic-based assessment) [7-10] or hand gestures movement (motion-based assessment) [11-13] during eating episodes. The majority of the proposed methods rely on single sensing approaches, for example, using electromyography sensor, accelerometer sensor, or microphone [14-17]. However, it is believed that precisely discriminating eating episodes from other confounding activities requires processing multiple parameters from several sources. For this reason, multimodal assessment is a common target of interest today. Taking as an example, using data from both accelerometer and gyroscope sensors proposed in [18] reached an accuracy of 75% in detecting eating activity. These kinds of sensors quantify specific features of hand-to-mouth gestures as well as jaw motion associated with dietary intake. Later on, adding images of food into data captured by accelerometer and gyroscope was suggested for eating episodes detection [19]. Analysis of these meal images can also extract information of food content and estimate dietary energy intake. Data taken by GPS were also added as input parameters to correctly predict eating activity [20]. Audio signals of chewing sound was a further option added to a data set of both motion data and meal images which improve the accuracy of eating periods detection up to 85% [21]. Therefore, according to the aforementioned studies, it is valuable to develop algorithms that can take advantage of multiple data sources for monitoring applications rather than focusing on a single sensor. The sources of different modalities will provide richer information in comparison to a single source.

However, although using different modalities provides further opportunities to explore more complementary information, the growing number of different modalities has brought new challenges due to increase in the volume and complexity of the data. For dealing with these high-dimensional data sets and lowering the computational time, some studies implement feature selection process including forward features selection [22], random forest [23], and principal component analysis [24] to reduce the data size and select important parameters/features. For combining the information captured by different sensors, the classification score fusion has been introduced in literature as an option. Papapanagiotou et al [25] fused support vector machine scores from both photoplethysmography and audio signals. Regarding discriminating eating episodes from other activities, researchers applied different classification tools from support vector machine [26] to artificial neural network [27]. They also found that appropriate epoch size ranges from 10 to 30 seconds [28]. However, recent advances in machine learning methods have increasingly captured the attention of many research groups for distinguishing food intake intervals from others based on deep learning techniques. Convolutional neural network has been used for automatically detecting intake gestures from raw video frames [29]. Convolutional neural network was also proposed by Ciocca et al [30] for image-based food recognition.

The aforementioned studies focused on data represented by features set and its corresponding fusion as well as decision fusion of classifiers. What remains to be addressed is investigating the sensor fusion for quantitatively integrating heterogeneous sources of information. Taking this into account, this study aimed to combine data derived from disparate sensors such that the resulting information has lower dimension and yet maintains the important aspects of original data.

To the best of our knowledge, there is no research focused on sensor fusion for personalized activity identification using different data sets collected by wearable devices. The proposed fusion here is based on a hypothesis that data captured by multiple sources are statistically correlated with each other and their 2D covariance representation has a unique distribution associated with the type of activity.

## Methods

### Implementation of Sensor Fusion Algorithm

The proposed algorithm automatically transformed data from different sensors in time into a single 2D color representation that provides fast effective support for discriminating eating episodes from others. The idea of this method was on the hypothesis that data driven by different sensors have correlation with each other and a covariance matrix of all these measurements has a unique distribution associated with each type of activity which can be visualized as a contour plot. With 2D covariance contour as input data sets, deep model–learned specific patterns in 2D correlation representation related to specific activity.

The following is a summary of the steps followed in the proposed method to detect eating episodes:

#### Step 1

Forming the observation matrix derived from all sensors; the corresponding covariance matrix can then be formed in 2 ways. The first way is to calculate pairwise covariance between each sample across all signals. The second way is to calculate pairwise covariance between each signal across all samples. The algorithm steps based on the second way are as follows:

The pairwise covariance calculation between each column combination:

C_ij_ = cov(H(:, i), H(:, j)) **(1)**

where H is observation matrix.

The covariance coefficient of 2 columns of i and j can be calculated as follows:

cov(S_i_, S_j_)=1/(n–1)Σ^m^_k=1_(S_ik_–µ_i_)(S_jk_–µ_j_) **(2)**

S_i_ = M(:, i), S_j_ = M(:, j)

where μ_i_ is the mean of S_i_, μ_j_ is the mean of S_j_, and m is the number of samples within the window.

#### Step 2

Obtaining the covariance coefficient matrix of all columns according to the following equation:



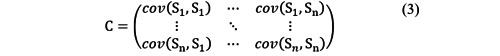



where n is the number of observations.

#### Step 3

Creating a filled contour plot containing the isolines of obtained matrix C so that given a value for covariance, lines are drawn for connecting the (x, y) coordinates where that covariance value occurs. The areas between the lines were then filled in solid color associated with the corresponding covariance value.

#### Step 4

Feeding contour plot to the deep network to classify the sequences related to each activity. Two different scenarios were considered for this study. These scenarios were different in terms of covariance matrix calculation, temporal segment size, type of activity, wearable device, subjects, and deep learning architecture. In the first scenario, calculating pairwise covariance between each sample across all signals was considered. In the second scenario, calculating pairwise covariance between each signal across all samples was taken into consideration.

### First Scenario for Evaluation of Algorithm

In the first scenario, data were recorded from a single participant wearing the Empatica E4 wristband on the right hand for 3 days. The data set includes the following data: (1) ACC—data from 3-axis accelerometer sensor in the range [–2g, 2g] (sampled at 32 Hz); (2) BVP—data from photoplethysmograph (sampled at 64 Hz); (3) EDA—data from the electrodermal activity sensor in microsiemens (sampled at 4 Hz); (4) IBI—interbeat intervals, which represent the time interval between individual beats of the heart (intermittent output with 1/64-second resolution); (5) TEMP—data from temperature sensor expressed in the °C scale (sampled at 4 Hz); and (6) HR—These data contain the average heart rate values, computed in spans of 10 seconds.

The window length for this scenario was selected to be 500 samples. This analysis was performed on 2954 500-sample segments after making all signals in the same size with sample frequency of 64 Hz. Segments were picked so that 1000 of them contained sleeping intervals, and 1000 of them captured during working with computer and others were during eating episodes.

**Figure 1:**
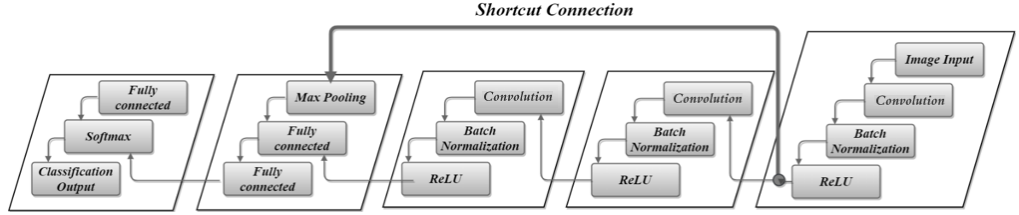
Deep learning network architecture.

The deep learning architecture used in this scenario was a deep residual network. The proposed deep learning architecture for image-to-label classification is presented in Figure 1 and consisted of a deep residual network with 3 2D convolution layers, followed by batch normalization, ReLU, max pooling, and fully connected layers. The 2D convolutional layer applied sliding convolutional filters to the input contour image. The output of this network is a categorical response, and therefore a softmax and classification layers were also added as last layers. There is also a shortcut to jump over some layers.

Table 1 provides detailed information about the proposed network layers. This information includes the sizes of layer activations. The training parameters of the deep learning model are given in Table 2. The mini-batch size and the maximum number of epochs were set to 100 and 10, respectively. Fivefold cross-validation was also used to check the performance of the model.

**Table 1:** Detailed information about the layers of proposed network.

Number	Layer type	Activations
1	Image input	300 × 300 × 3
2	Convolution	300 × 300 × 32
3	Batch normalization	300 × 300 × 32
4	ReLU	300 × 300 × 32
5	Convolution	150 × 150 × 64
6	Batch normalization	150 × 150 × 64
7	ReLU	150 × 150 × 64
8	Convolution	150 × 150 × 128
9	Batch normalization	150 × 150 × 128
10	ReLU	150 × 150 × 128
11	Convolution	150 × 150 × 128
12	Addition	150 × 150 × 128
13	Max pooling	75 × 75 × 128
14	Fully connected	1 × 1 × 500
15	Fully connected	1 × 1 × 10
16	Fully connected	1 × 1 × 3
17	Softmax	1 × 1 × 3
18	Classification output	—^a^

^a^—: Not available

**Table 2:** The model training parameters.

Parameter	Value
Initial learn rate	0.001
Learn rate drop factor	0.1
Learn rate drop period	2
Mini batch size	100
Max epochs	10
Learn rate schedule	Piecewise

### Second Scenario for Evaluation of Algorithm

In the second scenario, an open data set associated with the activities of daily living was considered. The data set was collected from 10 healthy participants, performing 186 activities of daily living while wearing 9-axis inertial measurement units on both left and right arms [31].

The considered activities can be grouped into 4 separate categories: (1) mobility, including walking, sitting down and standing up, and opening and closing the door; (2) eating, including pouring water and drinking from glass; (3) personal hygiene, including brushing teeth; and (4) housework, including cleaning the table [31].

The recorded data include quaternions (with resolution of 0.0001), accelerations along the x, y, and z axes (with resolution of 0.1 mG), and angular velocity along the x, y, and z axes (with resolution of 0.01 degrees per second) [31].

Data annotation for all the experiments was manually performed based on videos recorded by an RGB camera [31].

The window length for this scenario was selected to be 50 samples. This analysis was performed on 4478 50-sample segments. Segments were picked so that 1132 segments contained walking episodes, 20 segments contained sitting down episodes, 16 segments contained standing up episodes, 366 segments contained opening the door episodes, 400 segments contained closing the door episodes, 1208 segments contained pouring water and drinking from glass episodes, 704 segments contained brushing teeth episodes, and 632 segments contained cleaning the table episodes.

As training from the scratch on relatively small-scale data sets is susceptible to overfitting, a pretrained model for extracting deep features was suggested in this scenario. The deep learning architecture used in this section was the InceptionResNetV2 pretrained model. This pretrained classification network has already learned on more than 1 million images. As this network was trained on extremely large data sets, it is capable of being served as a generic model. Therefore, this section used layer activation of the pretrained InceptionResNetV2 architecture as features to train a support vector machine for classifying different activities. The parallel computing platform of Tesla P100 PCIe 16 GB was used for implementing this deep structure. The depth, size, and number of parameters in the pretrained InceptionResNetV2 network were 164, 209 MB, and 55.9 M, respectively. Leave-one-subject-out cross-validation was considered for performance evaluation of classification.

## Results

### Applying the Proposed Algorithm on the First Scenario

**Figure 2:**
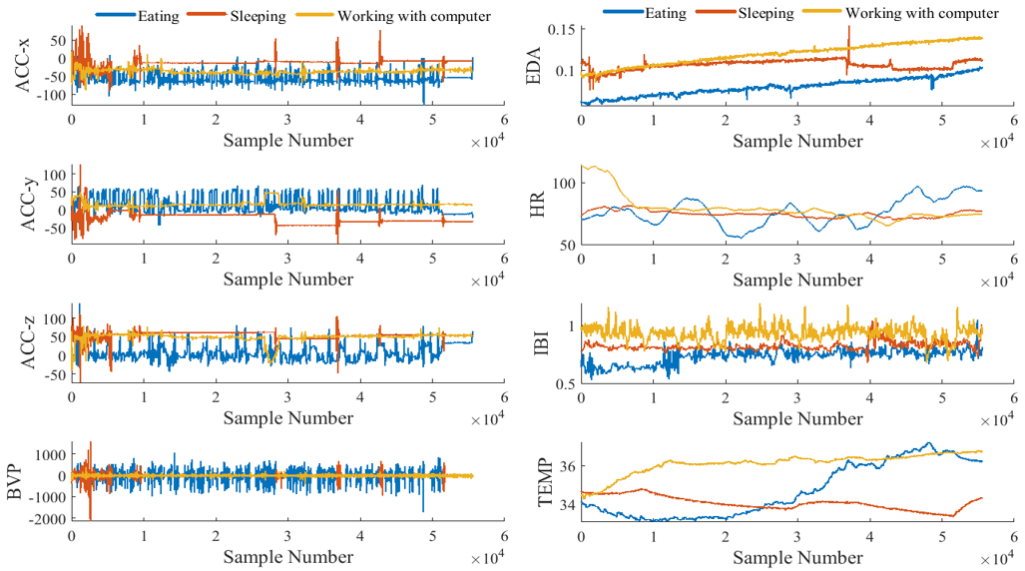
Time series amplitude of each modality captured during 1 episode of 3 different activities of eating, sleeping, and working with computer (the number of samples per second is 64).

**Figure 3:**
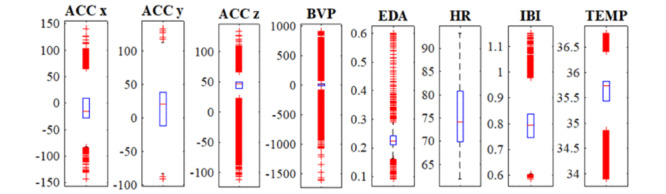
Distribution of data captured by different sensors for a portion of eating episode.

Signals captured from different sensors during eating, sleeping, and working with computer are plotted in Figure 2. This figure illustrates how characteristics of data coming from different sources vary a lot. Figure 3 shows how the values in the eating-related data captured by different sensors are spread out in their boxplots, and how their distributions differ from each other. As these signals cannot be described by the same distribution, they are said to be heterogeneous. This heterogeneity brings up the issue of how to integrate the information from such diverse modalities. This spread in the range of scales across the various modalities causes a simple approach to be not enough for reliable activity detection and a therefore a more sophisticated technique is required.

**Figure 4:**
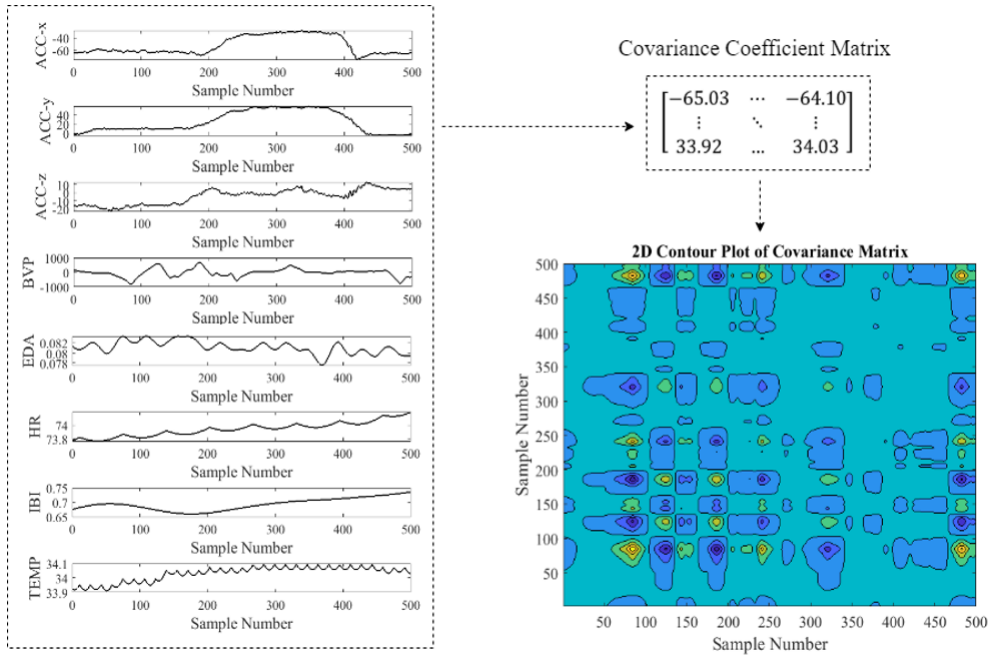
Generating covariance coefficients contour for a 500-sample eating episode (pairwise covariance was calculated between each sample across all signals).

**Figure 5:**
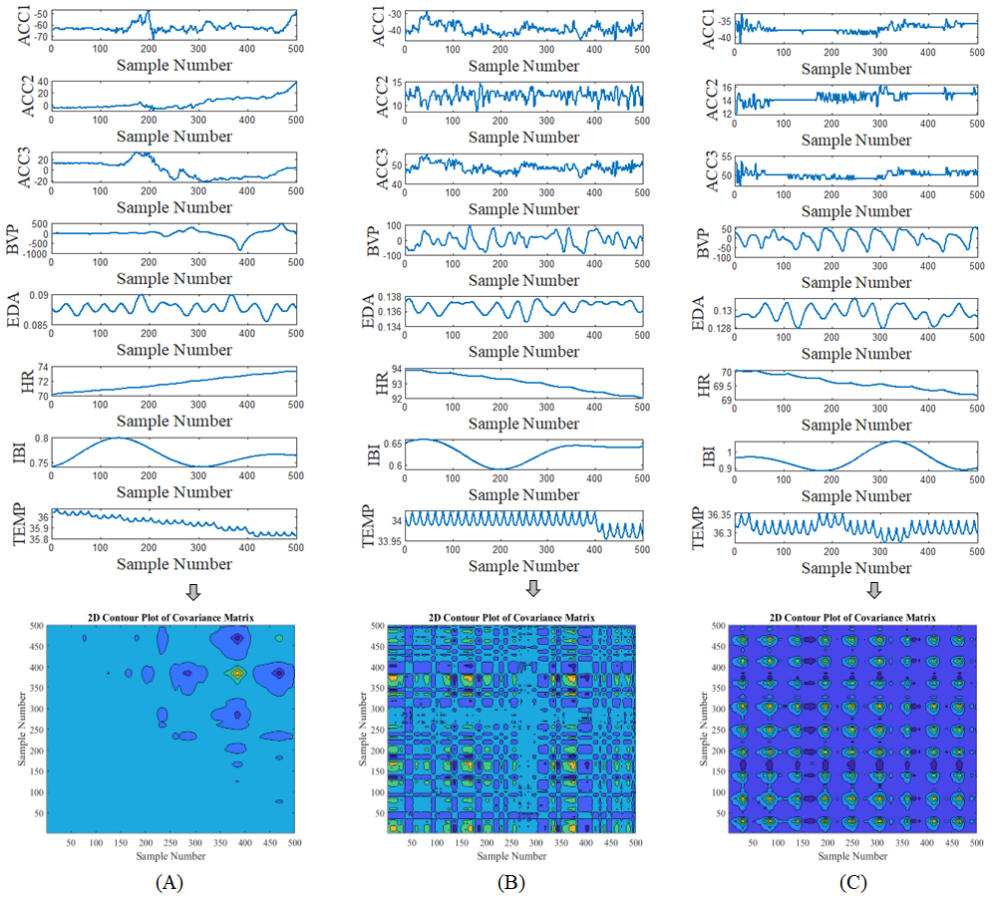
Examples of covariance coefficients contour for different activities. (A) Eating; (B) Working with computer; (C) Sleeping.

Figure 4 shows the preprocessing steps performed on the raw data to prepare input data for a 2D deep residual network. As shown in Figure 5, covariance coefficients between channels were first derived and presented in the form of contour map. The obtained image was fed to a deep network as input image.

Figure 5 shows examples of covariance coefficients contour generated from different modalities. The horizontal and vertical axes represent the sample number. The value of correlation coefficients is represented by the color, with dark colors corresponding to low values and bright colors corresponding to high values. As seen in figures, there is a visible difference in the color patterns of correlation coefficients contour for different activities.

**Figure 6:**
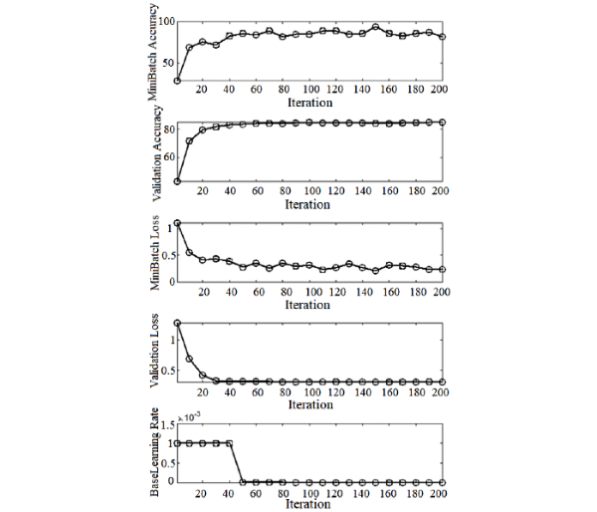
Deep learning model performance over observations in the mini-batch.

The performance metrics variations including epoch number, iteration number, time elapsed, mini-batch accuracy, validation accuracy, and loss function value for the validation data are plotted in Figure 6. The number of epochs was chosen to be 10 over 200 iterations. The training and testing proportions, being considered as 70% and 30%, respectively, were randomly assigned from each label. The training data were also shuffled before every epoch. Learning rate was reduced over epochs and its speed was updated by decreasing the learning rate, and multiplying it by a fractional learn rate drop factor over a specific number of epochs. According to Figure 6, the small validation loss allows to conclude that this method has generalization capability.

**Figure 7:**
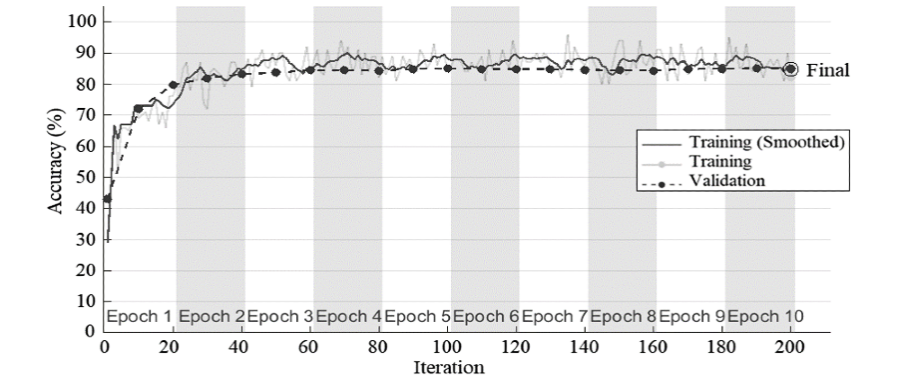
Accuracy variation in each epoch of deep residual network with input images of covariance coefficients contour.

**Figure 8:**
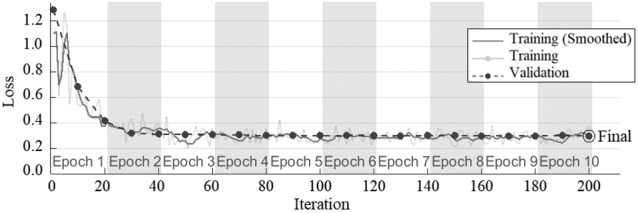
Loss function variation in each epoch of deep residual network with input images of covariance coefficients contour.

Convergence of average accuracy and loss function during training and validation for 10 epochs was plotted in Figures 7 and 8. The result of the covariance-based model has rapidly converged to a stable value with no sign of overfitting.

**Figure 9:**
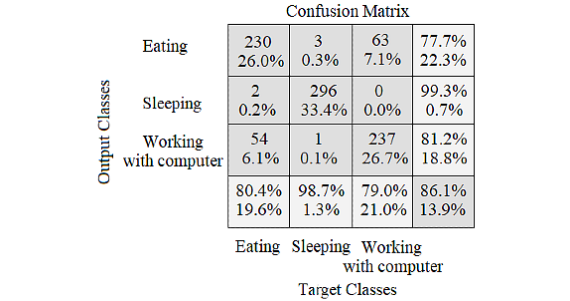
Confusion matrix for the validation set of deep residual network with input images of covariance coefficients contour.

Confusion matrix in Figure 9 shows the results obtained from validation data sets of covariance coefficients contour.

### Applying the Proposed Algorithm on the Second Scenario

**Figure 10:**
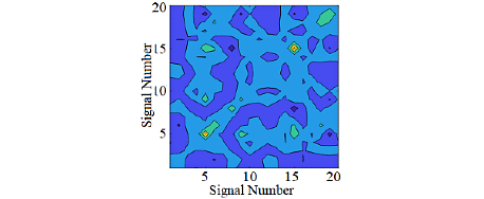
Covariance map for a 50-sample walking episode (pairwise covariance was calculated between each signal across all samples was taken into consideration).

Figure 10 shows an example of covariance map generated from different modalities in the second scenario.

**Figure 11:**
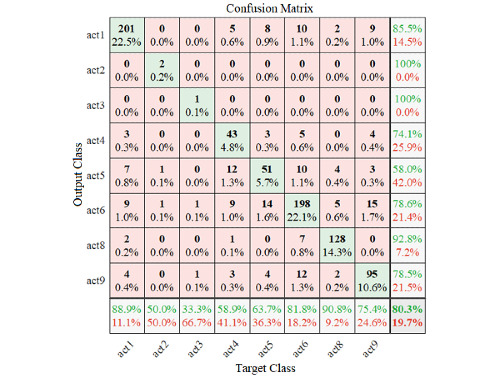
The results of applying pretrained deep learning architecture on the fused data.

The visualization of performance of the fusion method applied on the second scenario is plotted in Figure 11 and shows how the algorithm is confusing 2 classes.

The elapsed time for the training process of the model was 39.2714 seconds. The latency for making decision on the new input was 0.1459 seconds. Various statistics calculated from the confusion matrix for a comprehensive study are listed in Table 3. Based on classification results, it is possible to find how well classification of different activities was done by taking advantage of the proposed sensor fusion.

**Table 3:** Comprehensive study of pretrained deep learning classifier’s performance.

Metrics of classifier’s performance	Values
Accuracy	0.95083
Precision (positive predictive value)	0.80335
False discovery rate	0.19664
False omission rate	0.02809
Negative predictive value	0.97190
Prevalence	0.12500
Recall (hit rate, sensitivity, true positive rate)	0.80335
False-positive rate (fall-out)	0.02809
Positive likelihood ratio	28.5965
False-negative rate (miss rate)	0.19664
True-negative rate (specificity)	0.97190
Negative likelihood ratio	0.20233
Diagnostic odds ratio	141.334
Informedness	0.77525
Markedness	0.77525
F-score	0.80335
G-measure	0.80335
Matthews correlation coefficient	0.77525

### Comparison With Similar Studies

Data captured by different sensors in order to detect eating intervals have been explored by many studies. These studies focused on analyzing several types of data captured by different sensors from accelerometers and gyroscopes to respiratory inductance plethysmography and oral cavity. However, as seen in Table 3, the number of modalities involved in detecting food intake intervals has been up to 2. Therefore, this study investigated whether eating event detection by simultaneous processing of 8 different modalities (eg, 3-axis accelerometer sensor, photoplethysmography, electrodermal activity sensor, interbeat intervals, temperature sensor and heart rate) is feasible. The obtained results showed an overall validation accuracy comparable to the approaches proposed earlier in the literature (Table 4). Furthermore, the proposed data fusion framework in this research provided a simple way of integrating multiple data sources applicable for deep learning methods in human activity monitoring, while previous studies focused on applying raw data. When it comes to cloud computing as well as big data for the purpose of human activity monitoring using wearable sensor-based technologies, the cost of directly applying high-dimensional raw data to a deep classifier would be computationally expensive.

**Table 4:** Comparison of previous studies on food intake episodes detection.

Study	Modalities	Method	Accuracy, %
[32]	Acoustic signal	Correlation matching	85
[33]	Food image and speech recording	Support vector machine classification	90.6
[34]	Electroglottograph	Artificial neural network	86.6
[35]	Piezoelectricity	Time and amplitude thresholding	86
[36]	Accelerometer and gyroscope	Decision tree classifier	85.5
[37]	Chewing sound	(1) Deep Boltzmann and (2) Machine with deep neural network classifier	77
[38]	Piezoelectricity	Convolutional neural network	91.9
[39]	Acceleration and orientation velocity	Convolutional-recurrent neural network	82.5-96.4

### Investigating the Effect of Missing Values

The proposed data fusion technique also has the challenge of data imperfection, which could be overcome by using data imputation methods. Missing samples can affect the contour representation of covariance matrix to a great extent which makes it necessary to be recovered using missing value filling methods.

**Figure 12:**
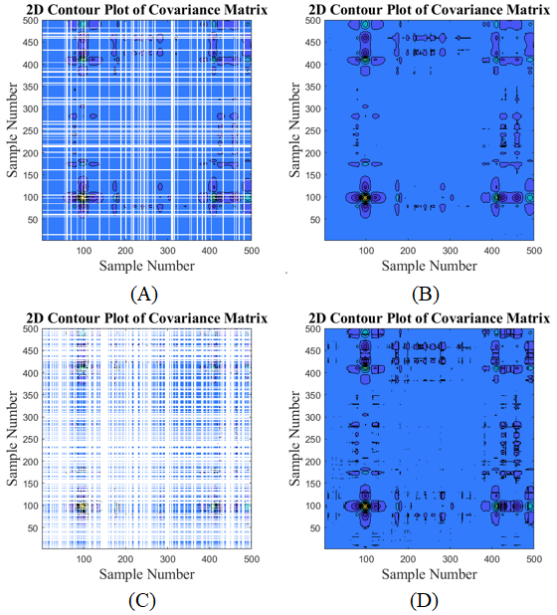
Contour representation (A and C) before and (B and D) after applying moving median for missing data.

Figure 12 demonstrates how the moving median method (as a sample of interpolation methods) can recover the contour representation suffered from the missing data. The moving median was done over a window of length 20. However, there is still a huge gap for addressing the issue of data inconsistencies, which will let the gates open for future studies.

Table 5 shows the impact of missing data without using any imputation on performance degradation in the second scenario.

**Table 5:** Impact of missing data on performance degradation.

Metrics of classifier’s performance	Missing value percentage					
	0	1.11	2.22	8.33	15.5	25
						
Accuracy	0.95083	0.91400	0.89591	0.89533	0.88501	0.88326
Precision (positive predictive value)	0.80335	0.65603	0.58365	0.58132	0.54007	0.53307
False discovery rate	0.19664	0.34396	0.41634	0.41867	0.45992	0.46692
False omission rate	0.02809	0.04913	0.05947	0.05981	0.06570	0.06670
Negative predictive value	0.97190	0.95086	0.94052	0.94018	0.93429	0.93329
Prevalence	0.12500	0.12500	0.12500	0.12500	0.12500	0.12500
Recall (hit rate, sensitivity, true-positive rate)	0.80335	0.65603	0.58365	0.58132	0.54007	0.53307
False positive rate (fall-out)	0.02809	0.04913	0.05947	0.05981	0.06570	0.06670
Positive likelihood ratio	28.5965	13.3506	9.81308	9.71933	8.21996	7.99166
False-negative rate (miss rate)	0.19664	0.34396	0.41634	0.41867	0.45992	0.46692
True-negative rate (specificity)	0.97190	0.95086	0.94052	0.94018	0.93429	0.93329
Negative likelihood ratio	0.20233	0.36174	0.44267	0.44531	0.49226	0.50029
Diagnostic odds ratio	141.334	36.9063	22.1678	21.8259	16.6982	15.9738
Informedness	0.77525	0.60689	0.52418	0.52151	0.47437	0.46637
Markedness	0.77525	0.60689	0.52418	0.52151	0.47437	0.46637
*F*-score	0.80335	0.65603	0.58365	0.58132	0.54007	0.53307
G-measure	0.80335	0.65603	0.58365	0.58132	0.54007	0.53307
Matthews correlation coefficient	0.77525	0.60689	0.52418	0.52151	0.47437	0.46637

## Discussion

### Principal Findings

Recent advances in biosensor technologies [40,41] and consumer electronics have led to precise physiological monitoring and more specifically accurate activity recognition. Activity recognition based on wearable device is one of the most rapidly growing research areas in personalization of analyses. Physical characteristics, health state, lifestyle, moving style, and gender are parameters that can be highly personalized. Therefore, in order to consider generalization of prediction or classification models, the data should be labeled personally, and the focus of research should be more on personalized analysis [42,43]. One way to personalize data is automatic identification of human activities and consequently labeling data based on different activities.

Regarding human activity recognition, we are facing upcoming transition from analyzing single modality to processing data collected from multiple sources with enormous diversity in terms of information, size, and behavior. This increases the complexity of classification problems and requires low-level data fusion to simultaneously integrate significant information in all modalities, and yet compressing the data directly at the source. This fusion process is important in a sense that reduction of communication load to other device or to the cloud requires local extraction of information from raw data stream in the sensor level. Therefore, fused raw data in a compressed form are super important in terms of minimizing the amount of data needed to be stored or needed to be sent. It is also important in saving battery power and reducing transmission time.

Regarding the importance of implementing a low-level fusion method with simple structure, this study presented a general framework for implementing efficient fusion based on covariance map. The promising classification results reached precision of 80.3% and showed that global 2D covariance representation can reliably quantify the difference between activities, as it provides a simple abstract representation of correlation over modalities.

For performance assessment of the proposed algorithm, the method was implemented on 2 separate scenarios. These scenarios were different regarding the temporal segment size, type of activity, wearable device, subjects, and deep learning architecture. The obtained results showed the ability of the proposed fusion technique to generalize to other data sets with different modalities, participants, and tasks.

### Limitations in Existing Literature

There are many ways of integrating modalities for activity recognition task [44-57], with the 3 major groups being sensor-level [45,49,50], feature-level [44,57], and decision-level [51-56] fusion. Fusion at the decision level is the most frequently used method which takes advantage of training machine learning and deep learning models for each modality. When it comes to merging scores of these networks for the purpose of data fusion, the practical applications are limited by their complex process which lead to more computationally heavy processing and make them inapplicable for implementing on low-power systems. Therefore, such techniques cannot be considered as low-level data fusion.

There are only a few methods for low-level data fusion, with 2 focused on using Bayesian network for sensor-level fusion [45,50]. These Bayesian networks usually involve a time-consuming process of hyperparameter tuning. Correctly implementing hyperparameter optimization is usually complex and computationally expensive. A small change in the values of hyperparameters can highly affect the performance of model. In this sense, it could be said that the low-level fusion methods with simple structure can outperform sophisticated ones.

### Strengths of the Proposed Method

Unlike complex fusion techniques based on evolutionary computation [48], machine learning approaches [47,51,53,54], Bayesian models [44,45,50,55], Kalman filtering [49], and neural networks [46], the proposed fusion method had very simple implementing procedure, and yet capable of revealing the common trends and similarities among recorded modalities. It was also free of the number and type of sensors used for collecting data. This could be an important benefit as there is high diversity in sensor technology deployed for activity recognition and the choice of sensors vary a lot from one case to another. The sensors used in activity recognition studies include vibration and contact sensor [44], tap sensor [45], motion sensor [46], ventilation sensor [47], heart rate sensor [48], magnetometer sensor [49], temperature sensor [50], electrocardiogram sensor [51], accelerometer sensor [52], and gyroscope [54]. Furthermore, this method is universal in a sense that it can cover a wide spectrum of problems including tracking activity of daily living, elderly monitoring, fall detection, smart home, ambient assisted living, behavior analysis, among others. It could help in decreasing the final cost of the monitoring framework by deploying fusion in the first step of classification process (applying fusion algorithm in the final step needs independently analyzing data for every single component and combining the final results which make the implementation computationally expensive). Providing the possibility of visually representing the correlation among modalities and reducing dimension by embedding the sensory data in just a single 2D representation can be considered as other strengths of the studied technique.

### Future Work

A limitation to this research is that both tested scenarios were performed on healthy volunteers, which may be far from the cases including actual patients who suffer from movement disability or major health problem. This could be included in future work.

Future research direction will also include implementing the fusion algorithms for the scenarios in which one or more modalities are missing. The applicability of the findings will be also tested for other problems rather than activity recognition.
